# HOXC10 promotes esophageal squamous cell carcinoma progression by targeting FOXA3 and indicates poor survival outcome

**DOI:** 10.1016/j.heliyon.2023.e21056

**Published:** 2023-10-14

**Authors:** Xiaoting He, Huiyu Wang, Runjie Wang, Yuting Li, Suqing Li, Xiufeng Cao, Junying Xu

**Affiliations:** a Oncology Department, The Affiliated Wuxi People's Hospital of Nanjing Medical University, Wuxi People's Hospital, Wuxi Medical Center, Nanjing Medical University, Wuxi, Jiangsu, 214023, China; b General Surgery Department, Nanjing First Hospital, Nanjing Medical University, Nanjing, Jiangsu, 210012, China

**Keywords:** HOXC10, FOXA3, Esophageal squamous cell carcinoma

## Abstract

**Background:**

Esophageal cancer is one of the most unknown and deadliest cancers in the world. Although recent studies have identified some mutations linked to the development of squamous cell carcinoma of the esophagus (ESCC), the specific role of HomeoboxC10 (HOXC10) in the pathogenesis still requires further investigation.

**Methods:**

Agilent mRNA single-channel gene expression was employed to identify genome-wide gene signatures in ESCC. These signatures were also verified using qRT-PCR, immunohistochemical staining as well as Western blot. The biological functions of HOXC10 were further investigated through cellular studies conducted on ESCC cells. Survival analysis was conducted utilizing the Kaplan-Meier method. The GEPIA database and the STRING website were utilized to predict the potential targets that bind to HOXC10. Co-immunoprecipitation assays were performed to investigate the binding interaction between HOXC10 and Forkhead Box A3 (FOXA3). Animal models were established to analyze the effects of HOXC10 silencing on tumorigenesis *in vivo*.

**Results:**

The expression levels of HOXC10 mRNA were found to be upregulated in ESCC. Survival analysis revealed a significant association between abnormally elevated levels of HOXC10 mRNA and an unfavorable prognosis in patients with ESCC. *In vitro* studies revealed that the knockdown of HOXC10 expression resulted in the inhibition of the proliferation, invasion, and migrating ability of ESCC cells through the upregulation of FOXA3. Furthermore, tumor-bearing mouse models studies demonstrated that HOXC10 through knockdown techniques significantly suppressed ESCC tumor growth. HOXC10 was found to enhance the activation of the MAPK signaling pathway by regulating FOXA3 in ESCC cells.

**Conclusion:**

These results support a key role for HOXC10 in the tumorigenesis of ESCC by upregulating FOXA3 via the MAPK pathway and highlight its potential as a promising diagnostic and prognostic marker for ESCC.

## Highlights

• HOXC10 is upregulated in ESCC and linked with prognosis.
• HOXC10 facilitates the migration and invasiveness and inhibits apoptosis of ESCC cells.
• HOXC10 upregulates FOXA3 to activate the MAPK signaling in ESCC cells.
• HOXC10 promotes ESCC tumorigenesis in vitro and *in vivo*.

## 1 Introduction

Esophageal cancer (EC) is the seventh most frequently diagnosed malignancy and ranks sixth in cancer mortality across the world in 2020 [1,2]. esophageal squamous cell carcinoma (ESCC) is the most prevalent histological subtype of EC, accounting for 90 % of EC patients in high-risk regions [3,4]. Life and environmental factors, such as smoking, alcohol overconsumption, and hot beverage consumption, increase the risks of ESCC [3,5]. Currently, the treatment options for ESCC mainly include surgery, radiotherapy, chemotherapy, and targeted therapy [6]. The most frequent presenting symptom of EC is dysphagia, either by itself or in conjunction with unintended weight loss. However, EC are often asymptomatic and easily neglected at the early stages, and patients are often found at the advanced stage accompanied by lymph node metastasis, with a 5-year survival rate of only around 20 % [7,8]. Therefore, exploring novel therapeutic targets may help in developing effective strategies for treating ESCC and improving the prognosis.

HomeoboxC10 (HOXC10), a highly conserved transcription factor, is crucially involved in embryonic morphogenesis during development, and exploits DNA replication and embryonic capacity for tumor development [[9], [10], [11], [12]]. Substantial literature has shown that HOXC10 acts as a tumor-promoter in the occurrence and development of many solid tumors [13]. For example, HOXC10 promots migration, Epithelial-mesenchymal transition (EMT) as well as tumorigenesis in ovarian cancer by promoting the transcription of Slug [14]. HOXC10 encourages the invasion and migration of thyroid cancer cells, suggesting its potential significance as a novel biomarker for predicting the prognosis of human thyroid cancer. It also encourages the metastasis of human lung adenocarcinomas and denotes a bad prognosis for survival [15,16]. HOXC10 is a direct target of miR-136, which prevents the peritoneal spread of gastric cancer. This suggests that HOXC10 promotes metastasis in peritoneal metastases of gastric cancer [17]. HOXC10 overexpression has been reported to facilitate the proliferation, migration, and angiogenesis of HUVECs, and is revealed to induce angiogenesis in gliomas by upregulating vascular endothelial growth factor A (VEGFA) [18]. A study also indicated that HOXC10 positively regulated the proliferation, migration, and invasiveness of gastric cancer by elevating proinflammatory factor expression through NF-κB signaling [19]. HOXC10 dysregulation has been demonstrated to be closely associated with ESCC development, and HOXC10 overexpression drives ESCC cell growth and enhances the resistance to chemoradiotherapy in ESCC [20]. Whereas, the role of HOXC10 in ESCC and the underlying mechanism are still not fully elucidated.

Forkhead box A3 (FOXA3) belongs to the Forkhead-box (FOX) gene family, the dysregulation of which is responsible for various human diseases such as congenital disorders, diabetes mellitus, and carcinogenesis [21,22]. FOXA3 upregulation is identified in the esophageal cancer samples, and its level is positive correlated with invasion and metastasis in EC. Additionally, FOXA3 silencing is revealed to suppress the migrating ability and invasiveness of EC cells [23]. FOXA3 is also revealed to promote the proliferation as well as invasion of cholangiocarcinoma cells [24]. Disheveled binding antagonist of beta catenin3 antisense1 (DACT3-AS1) interacts with FOXA3 to promote cell migration, invasiveness, and EMT in hepatocellular carcinoma [25]. Based on the bioinformatics analysis, FOXA3 is a similar gene to HOXC10, and their expression is positively correlated in esophageal cancer.

In our study, the gene expression profile in five ESCC tissue samples and adjacent non-cancerous tissue (NT) was explored using an Agilent single-channel expression profile chip. HOXC10 was differentially up-regulated in ESCC, and the biologic effects and underlying mechanism of HOXC10 on ESCC development were further elucidated. We assumed that HOXC10 facilitated ESCC malignancy by interacting with FOXA3. The findings of this study might deepen the understanding of the role of HOXC10 in ESCC and provide new insights into targeted therapy for ESCC.

## 2 Materials and methods

### 2.1 Study population

Paired paraffin-embedded ESCC and adjacent normal tissue (NT) samples were obtained from patients recruited during radical surgery at the Affiliated Nanjing First Hospital of Nanjing Medical University, in 2011–2012 (n = 81), and the Affiliated Wuxi People's Hospital of Nanjing Medical University, in 2017–2018 (n = 10). The diagnosis of cancer was confirmed by pathology. No patient received antitumor therapy prior to undergoing radical surgery. NT was defined as tissue at least 2 cm away from the tumor edge. The patient's clinicopathological information was presented in Table 1. ESCC samples were classified according to the American Joint Cancer Committee (AJCC) [26] classification guidelines. The classification and histopathology subtyping of the ESCC samples were based on criteria of the World Health Organization (WHO) [27]. The patients were followed up regularly. The study has obtained approval from the Ethics Committee of the Nanjing Medical University Cancer Centre (Nanjing, China). All patients signed the informed consent.

**Table 1:** Association between HOXC10 expression and esophageal squamous cell carcinoma clinicopathological characteristics.

Characteristics	Number	HOXC10 mRNA expression		*p*
		High (%)	Low (%)	
Age, years				0.441
<60	33	15(36.59)	18(45)	
≥60	48	26(63.41)	22(55)	
sex				0.54
Male	52	25(60.98)	27(67.5)	
Female	29	16(39.02)	13(32.5)	
Cell differentiation				0.19
well	12	4(9.76)	8(20)	
moderate	58	29(70.73)	29(72.5)	
poor	11	8(19.51)	3(7.5)	
Tumour invasion depth				0.006
T1	7	3(7.32)	4(10)	
T2	23	6(14.63)	17(42.5)	
T3	50	31(75.61)	19(47.5)	
T4	1	1(2.44)	0(0)	
Lymph node metastasis				0.202
N0	42	18(43.9)	24(58.54)	
N1	31	17(41.46)	14(35)	
N2	8	6(14.64)	2(5)	
N3	0	0	0	
TNM stage				0.006
Ⅰ	6	2(4.88)	4(10)	
Ⅱ	44	17(41.46)	27(67.5)	
Ⅲ	31	22(53.66)	9(22.5)	
Ⅳ	0	0(0)	0(0)	

*P-values were calculated using Pearson's χ2 test or Fisher's exact test, as appropriate.

### 2.2 Agilent mRNA single-channel expression profiling

An EasyPure RNA kit (TransGen Biotech, Beijing, China) was applied to collect total RNAs from tumor paraffin blocks and matched NTs. The collected RNAs were then purified and fluorescently labeled using a Crystal Core® cRNA amplification marker kit (Department of CapitalBio Corporation, Beijing, China), following the manufacturer's protocol. The Agilent G2565CA microarray scanner was applied to scan the chip for hybrid images, which were then subjected to the feature extraction image analysis software. After inputting the raw data into the GeneSpring GX software, the percentile shift method was applied for signal normalization. The differentially expressed RNAs were identified by absolute fold change of 2.

### 2.3 Quantitative RT-PCR (qRT-PCR)

RNA was collected from tissues and cells using Trizol according to the operating instructions. Subsequently, cDNAs were synthesized following the protocol provided by the producer (Invitrogen). Next, qRT-PCR was performed with SYBR Green II (Takara)was and then conducted under the condition of denaturation at 95 °C for 30 s, then 40 cycles of denaturation at 95 °C for 60 s and annealing for 34 s at 60 °C, and an extension at 95 °C for 15 s, 60 °C for 60 esc, and 95 °C for 5 s. Semiquantitative PCR was conducted with the StepOnePlusTM software v2.0 (Bio-Rad, Hercules, CA, USA) on the ABI 7500 Real-time PCR system. Relative RNA expression was evaluated with the 2^−ΔΔCt^ method, and GADPH was used as an internal reference. Primer sequences were presented below.

**Table:** 

	F (5′-3′)	R (5′-3′)
HOXC10	ACCACAGGAAATTGGCTGAC	GATCCGATTCTCTCGGTTCA
FOXA3	CCTCACTCCTGAATCACCC	GGTCATGTAGGAGTTGAGGG
GAPDH	TCATTTCCTGGTATGACAACGA	GGTCTTACTCCTTGGAGGC

### 2.4 Immunohistochemistry (IHC)

Briefly, 5-μm thick sections of ESCC patient or mouse tissue were cut from paraffin blocks and mounted on microscope slides. After deparaffinization and rehydration, the slides were then blocked and maintained with anti-HOXC10 (ab153904, 1:100, Abcam), anti-Ki-67 (ab15580, 5 μg/mL, Abcam), and anti-FOXA3 (PA5-106980, 0.2–1 μg/mL, Thermo Fisher) overnight at 4 °C. Rabbit IgG polyclonal-Isotype control (ab172730, 1:100, Abcam) was used as a negative control. Subsequently, slices were cultured with the corresponding secondary antibodies (ab205718, 1:500, Abam) for 30 min. Lastly, the slices were dyed with DAB solution for 5 min. Slices were then observed through the light microscope (Olympus). H-score method was used for stain intensity quantification and was calculated as previously reported. H-score lower than 200, IHC negative indicated low HOXC10 expression and H-score≥200, IHC positive indicated high HOXC10 protein expression.

### 2.5 Cell culture

Immortalized normal esophageal epithelial cell line (Het-1A) and human ESCC cell lines (TE10, KYSE140, KYSE150, KYSE180, KYSE410, KYSE450, KYSE30, and KYSE70) were purchased from the American Type Culture Collection (ATCC). All cell lines were cultured in RPMI-1640 (Invitrogen) supplemented with 10 % FBS and 1 % penicillin/streptomycin at 37 °C in a humidified 5 % CO_2_ incubator. KYSE140 and TE10 were selected for this study from the RT-PCR findings, which showed 10-fold higher expression of Hoxc10 mRNA than in Het1A cells.

### 2.6 Plasmid construction and transfection

The HOXC10 ORF (spanning nucleotides 99–1127, GenBank Accession Number NM_017409) was selected for the RNAi target sequence. Two shRNA primers were designed (http://bioinfo.clontech.com/rnaidesigner/) and synthesized by the GENEWIZ (Suzhou, China, Headquartered in New Jersey, USA): shHOXC10-1F: 5′-gatccGTGTCAAGGAGGAGAATGTTTCAAGAGAACATTCTCCTCCTTGACACTTTTTTg-3'; shHOXC10-1R: 5′-aattcAAAAAAGTGTCAAGGAGGAGAATGTTCTCTTGAAACATTCTCCTCCTTGACACg-3'; shHOXC10-2F: 5′-gatccGCAAGACCATTAACCTTACTTCAAGAGAGTAAGGTTAATGGTCTTGCTTTTTTg-3'; shHOXC10-2R: 5′-aattcAAAAAAGCAAGACCATTAACCTTACTCTCTTGAAGTAAGGTTAATGGTCTTGCg-3'. The pGreenPuro™ shRNA Expression Lentivector (SBI, Cat. #SI505A-1) was synthesized following the manufacturer's protocol, which including cloning shHOXC10 in pGreenPurTM Vector, ligating it into the linearized pGreenPuro™ lentivector, identifying clones containing the target shHOXC10, and purifying the shHOXC10 lentivector construct. For FOXA3 overexpression, pcDNA3.1/FOXA3 and pcDNA3.1 empty vectors were provided by GeneChem (Shanghai, China). Selected cell lines (KYSE140 and TE10) were transfected with the indicated plasmids, and the efficiency of silencing or overexpression was analyzed using qRT-PCR or western blotting, and stably transduced cells were selected for follow-up tests.

### 2.7 Western blot

Total proteins in tumor samples or ESCC cells were extracted with RIPA lysis buffer (Sigma-Aldrich) and then measured using a Micro BCA Protein Assay Kit (Pierce Biotechnology). Protein samples were loaded on 12 % SDS-PAGE, and then electro-transferred to PVDF membranes. Immunoblotting was conducted with anti-HOXC10 (ab153904, 1/2000; Abcam) and anti-FOXA3 (PA5-106980, 0.2–1 μg/mL, Thermo Fisher) at 37 °C for 2 h. The blots were then incubated with the corresponding secondary antibodies (ab205718, 1:10,000, Abcam) for 60 min at ambient temperature. GAPDH (ab9485, 1/1000; Abcam) was applied as an internal reference. The ChemiDoc image analysis system (Bio-Rad, USA) was used to analyze and quantify relative protein levels.

### 2.8 In vitro cell invasion tests

Transwell chambers (Millipore, Billerica, MA, USA) with 8-mm pore inserts were coated with 200 μL Matrigel in serum-free medium. Transfected cells (4 × 105) were seeded in the upper chamber with a Matrigel-coated membrane and allowed to invade through the membrane into the lower chamber of a 24-well plate filled with RPMI1640 with 10 % FBS. After 24–48 h of incubation, the non-invading cells were removed from the upper chamber surface. The cells invaded into the lower chamber were fastened and dyed using crystal violet, then the images were photographed by digital microscopy, and the invaded cell number was calculated manually.

### 2.9 Wound healing assays

The treated ESCC cells were inoculated into a six-well plate and maintained to reach 95 % confluency. Then a 200 μL suction pipette tip (approximately 1 mm wide) was used to scratch the cell monolayers, followed by PBS rinsing three times. Then cells were incubated in complete medium at 37 °C. To evaluate the ESCC cell migration in each group, a microscope was applied to capture images at 0h and 24h.

### 2.10 Cell proliferation

Cell Counting Kit-8 (CCK-8) (C0037, Beyotime Biotechnology, China) was used to evaluate cell proliferation following the manufacturer's instructions. Cells were seeded in 96-well plates at the confluence of 2000 cells per well for 24 h. They were then transfected with sh-RNA and subsequently incubated for 24, 48, 72 and 96 h. One hour before the end of each incubation period, 10 μl of CCK-8 reagent was added to each well. Stained cells were analyzed for absorbance at a wavelength of 450 nm using a fluorescence microscope. Continuous testing was conducted for 5 days.

### 2.11 Cell apoptosis

Annexin V-FITC/PI Kit (C1062 M, Beyotime Biotechnology, China) and the FACSCalibur flow cytometer were applied to determine the changes in apoptosis. In brief, transfected ESCC cells were collected and rinsed twice with PBS, followed by resuspension in PBS (10^5^ cells/mL). Next, fluorescein isothiocyanate (FITC) labeled Annexin V and propidium iodide (PI) were supplemented and cultured for 15 min avoiding light. Finally, the apoptosis of ESCC cells in each group was analyzed by a FACSCalibur flow cytometer.

2.12 Co-immunoprecipitation (co-IP) assays.

Total proteins from ESCC cells were collected using RIPA lysis buffer. For immunoprecipitation, protein samples (500 μg) were cultured with anti-HOXC10 (ab153904, 1/2000; Abcam), anti-FOXA3 (PA5-106980, 0.2–1 μg/mL, Thermo Fisher), and IgG (ab181236, 1:2,000, Abcam) overnight at 4 °C. Next, Protein G/A agarose beads (40 μl, Thermo Fisher) were supplemented into cell lysate and maintained for 2 h. After rinsing the beads with PBS thrice, precipitated proteins were resuspended in SDS-PAGE loading buffer, followed by boiling for 5 min and eluting proteins from the beads. The expression of FOXA3 and HOXC10 in the precipitates was subjected to western blotting.

### 2.12 Animal experiment

A total of ten female BALB/c nude mice (4–5 weeks old) were obtained from Vital River (Beijing, China). Mice were randomized into the shCtrl and shHOXC10-1 groups (5 mice in each group). For the establishment of the subcutaneous implantation model, mice received a subcutaneous injection of 1 × 10^6^ transfected TE10 cells into the flank. Tumor size was monitored every week, and the volume was calculated using formula V=(W^2^ × L/2). Mice were euthanized after 4 weeks by cervical dislocation, and tumors subsequently excised and weighed. The tumor tissues were collected and subjected to histological examination using HE staining.

### 2.13 Hematoxylin and eosin (HE)

The mouse tumor tissues were preserved at 4 °C while submerged in 0.1 mol/L PBS containing 4 % paraformaldehyde. Sections of tissues fixed in paraffin were cut into 5-μm thick pieces. Sections were stained with HE and examined under an optical microscope to determine how the morphological changes had changed.

### 2.14 Statistical analysis

Data were analyzed with GraphPad Prism 8.0 software and shown as the mean ± standard deviation. Statistical significance was evaluated via the Student's *t*-test, one-way analysis of variance (ANOVA), Pearson's χ^2^ test, Fisher's exact test, or regression analyses. The Kaplan-Meier analysis was used to estimate DFS and OS with the Log rank test. The multivariate analyses with the Cox proportional hazards model were conducted for prognostic factor identification. Gene expression correlation was subjected to Spearman correlation analysis. P < 0.05 was considered to be the threshold value.

## 3 Results

### 3.1 Identification of genes differentially expressed RNAs in ESCC

**Fig. 1:**
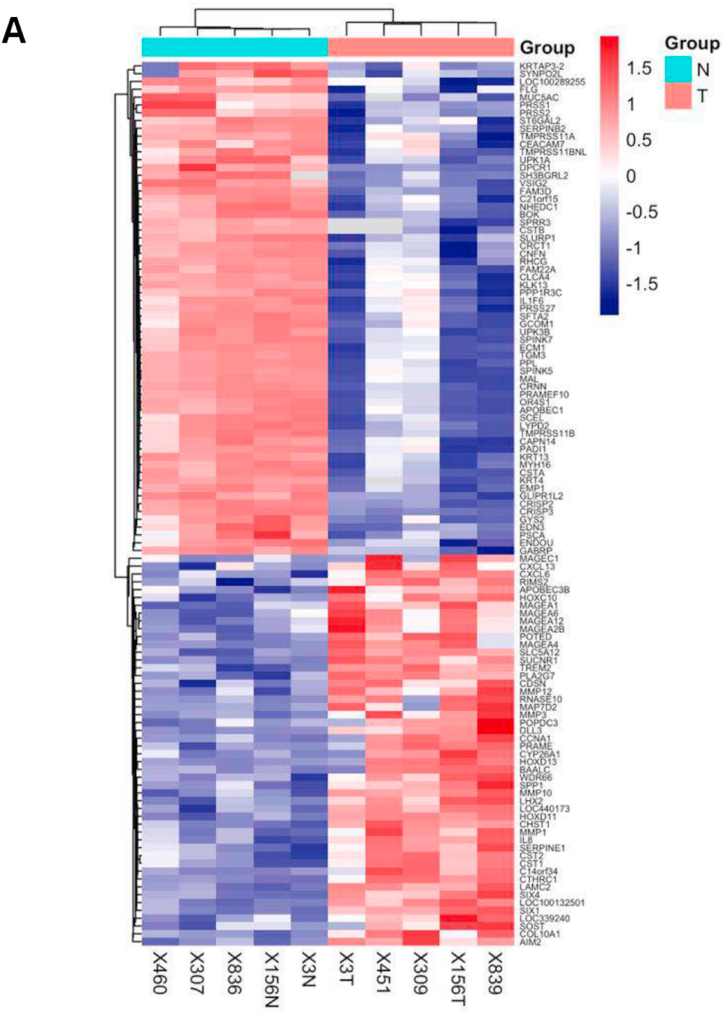
The differentially expressed genes in ESCC. (A) Gene expression signatures in 5 pairs of ESCC samples and adjacent NTs were explored using a single-channel Agilent mRNA expression profile chip.

The single-channel Agilent mRNA expression profile chip was applied for the gene expression profile in 5 pairs of paraffin-embedded ESCC specimens and adjacent non-cancerous tissues. The results showed that 77 genes were up-regulated at least 10-fold (p < 0.05) in the ESCCs compared to NTs (Fig. 1A). HOXC10 mRNA levels in ESCCs were 11.62-fold higher than those in adjacent NTs (*p* = 0.014885), which indicated that HOXC10 is involved in ESCC progression. Overall, HOXC10 mRNA expression is elevated in ESCC tissues and may play a role in the progression of ESCC.

### 3.2 Upregulation of HOXC10 in ESCC is related to poor prognosis

**Fig. 2:**
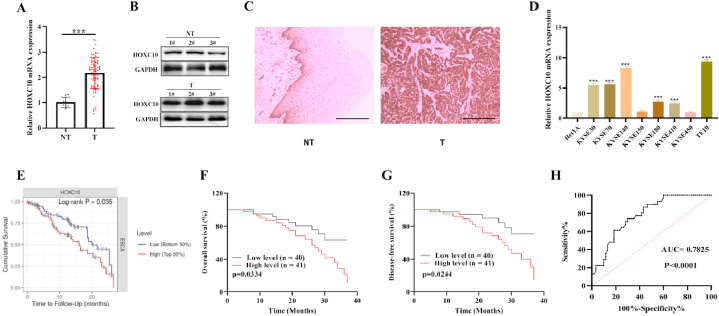
HOXC10 is upregulated in ESCC and linked with ESCC prognosis. (A) qRT-PCR measured the levels of HOXC10 mRNA in tumor samples (n = 81) and NTs (n = 10) from ESCC patients. (B) HOXC10 protein levels in 3 pairs of ESCC specimens and NTs were examined using western blotting. (C) IHC indicated that HOXC10 protein in ESCC was markedly higher expressed than in paired NTs and located in both the cytoplasm and nucleus, with a similar proportion (n = 3). (D) HOXC10 mRNA level in 8 esophageal squamous cell lines. (E) TIMER website (https://cistrome.shinyapps.io/timer/) conducted a survival analysis of EC patients with different levels of HOXC10. (F) OS and (G) DFS in ESCC patients with different HOXC10 levels was assessed using Kaplan-Meier analysis and the logarithmic test. (H) ROC curves for assessing the diagnostic value of HOXC10 in ESCC. All experiments were triplicated. Scale bar = 100 μm **P < 0.01, ***P < 0.001.

The expression of HOXC10 in collected ESCC patient samples was evaluated. We confirmed that the level of HOXC10 level was higher in the in 75 out 81 (92.59 %) paraffin-embedded ESCC tissues compared to adjacent NTs (*p* < 0.001) (Fig. 2A). Western blot also revealed a significant downregulation of HOXC10 in ESCC tissues (Fig. 2B). Moreover, according to the results of IHC assays (Fig. 2C), in 10 cases of ESCC samples, HOXC10 was found to be strongly positive (+++) in 9 samples and moderately positive (++) in one sample, with H-score >200 in all samples. However, in the 10 cases of NTs, HOXC10 protein was moderately positive (++) in 1 case, weakly positive (+) in 5 cases, negative (−) in 4 cases (H-score ＜ 200). In addition, the HOXC10 protein was localized in the cytoplasm in case 7 and in both the cytoplasm and nucleus in the remaining nine cases, with a similar proportion. Moreover, the upregulation of HOXC10 was also observed in 6/8 ESCC cell lines relative to Het1A cells (Fig. 2D). According to the survival analysis on the TIMER database, patients with ESCC who had high levels of HOXC10 were associated with adverse overall survival (Fig. 2E). The link of HOXC10 mRNA expression with patient clinicopathological characteristics was also analyzed in 81 ESCC patients. High HOXC10 level was correlated with higher tumor invasion depth (*P* = 0.006) and the later TNM stage (*P* = 0.006) (Table 1). The five-year survival in 81 patients with esophageal squamous cell carcinoma was 22.22 % (18/81). As exhibited in Fig. 2F and G, we have confirmed that ESCC patients with high HOXC10 level had significantly poorer overall survival (OS) (p = 0.0334) and disease-free-survival (DFS) outcomes (p = 0.0244). ROC curves indicated that the AUC value of HOXC10 in ESCC was 0.7825, suggesting that HOXC10 is a promising biomarker for ESCC diagnosis (Fig. 2H). Based on the results of Cox regression analysis, HOXC10, along with the TNM staging and cell differentiation independent prognostic factors for ESCC patients (Table 2). HOXC10 was significantly upregulated in ESCC tissues and was associated with a poor prognosis.

**Table 2:** Analyzes of prognostic parameters in patients with esophageal squamous cell carcinoma.

variable	Factors of layered	HR (95%CI)	*p*
Univariate analysis (Kaplan-meier analysis and the log-rank test)			
TNM stage	Ⅰ/Ⅱ/Ⅲ		<0.001
Cell differentiation	well/moderate/poor		0.025
Lymph node metastasis	N0/N1–N2		<0.001
Tumour invasion depth	T1/T2/T3		<0.001
Sex	male/female		0.374
Age (y)	<60/≥60		0.631
HOXC10 mRNA	low/high expression		<0.001
Multivariate analysis (COX regression analysis)		HR (95%CI)	
TNM stage	Ⅰ/Ⅱ/Ⅲ	6.368(2.298–17.641)	<0.001
Cell differentiation	well/moderate/poor	2.291（1.460–3.597）	<0.001
HOXC10 mRNA	high/lower expression	0.554(0.312–0.982)	0.043

HR, hazard ratio; CI, confidence interval.

### 3.3 HOXC10 facilitates the migration and invasiveness and inhibits apoptosis of ESCC cells

**Fig. 3:**
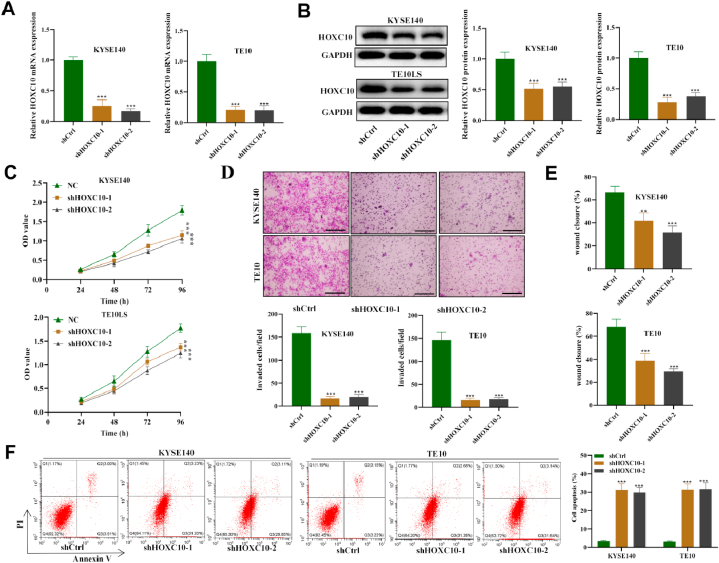
HOXC10 silencing reduces the migration and invasiveness and promotes apoptosis of ESCC cells. (A) qRT-PCR and (B) Western blot were used to assess the knockdown efficiency of HOXC10 in ESCC cell lines. (C) The viability of ESCC cells transfected with shCtrl or sh-HOXC10-1/-2 was analyzed by CCK-8 assays. (D) Transwell assays detected migration and invasiveness of ESCC cell in each group. (E) The migrating ability of ESCC cell was also assessed with wound scratch assays. (F) The impact induced by HOXC10 silencing on ESCC cell apoptotic rate was subject to flow cytometry. All experiments were triplicated. Scale bar = 50 μm *P < 0.05, **P < 0.01, ***P < 0.001.

Due to the highest expression level of HOXC10, TE10 and KYSE140 were selected for this study (Fig. 2D). Then we confirmed that both shHOXC10-1 and shHOXC10-2 significantly decreased HOXC10 mRNA and protein expression in ESCC cell lines compared to shCtrl (Fig. 3A and B). The ESCC cell growth was significantly inhibited after silencing HOXC10, suggesting that HOXC10 promotes the proliferative capacity of ESCC cells in vitro (Fig. 3C). Moreover, the migration and invasiveness was evaluated and migrated and invaded cell number and the wound closure distance showed a significant reduction in the shHOXC10-1/-2 groups relative to the shCtrl (Fig. 3D and E). As revealed by flow cytometry, HOXC10 knockdown induced a significant elevation in the apoptotic rate of ESCC cells relative to the shCtrl group. Overall, HOXC10 acts as an oncogene that enhances cell viability, migration, and invasiveness, while suppressing apoptosis in ESCC.

### 3.4 HOXC10 interacts with FOXA3 and upregulates FOXA3 expression in ESCC cells

**Fig. 4:**
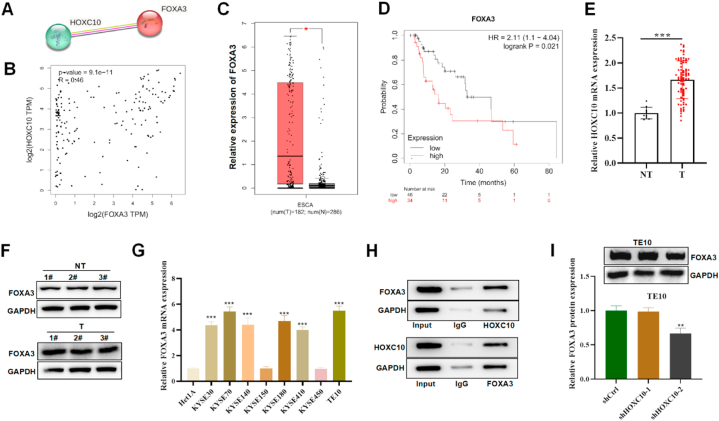
HOXC10 binds with FOXA3 in ESCC cells. (A) The interaction between HOXC10 and FOXA3 proteins is predicted by the String database. (B) GEPIA website showed a correlation between HOXC10 and FOXA3 in esophageal carcinoma. (C) GEPIA database presented the expression pattern of FOXA3 in esophageal carcinoma. (D) The survival curves of esophageal carcinoma patients with different FOXA3 expression on the Kaplan-Meier Plotter database (http://kmplot.com/analysis/). (E) qRT-PCR and (F) Western blot were used to measured FOXA3 expression in ESCC patient samples and NTs. (G) qRT-PCR detected FOXA3 mRNA level in ESCC cell lines. (H) Co-IP assays evaluated the biding of FOXA3 with HOXC10 in ESCC cells. (I) Western blot detected FOXA3 protein expression in ESCC cells with HOXC10-silenced. All experiments were triplicated. **P < 0.01, ***P < 0.001.

FOXA3 was predicted to be a similar gene to HOXC10 in esophageal cancer. Based on the STRING and GEIPA databases, FOXA3 and HOXC10 were co-expressed and their expression was positively correlated in esophageal cancer (R = 0.46, p = 9.1e-11) (Fig. 4A and B). The expression pattern of FOXA3 was analyzed on the GEPIA website, FOXA4 was evidently upregulated in EC (Fig. 4C). In addition, patients with esophageal cancer were also predicted to unfavorable survival with high levels of FOXA3 (HR = 2.11[1.1–4.04], P = 0.021) (Fig. 4D). Moreover, FOXA3 mRNA and protein were found to be upregulated in ESCC tumour samples relative to NTs (Fig. 4E and F). Similarly, higher expression of FOXA3 was also detected in ESCC cell lines relative to Het1A cells (Fig. 4G). As revealed by Co-IP assays, HOXC10 was found significantly enriched in the precipitates of anti-FOXA3 and FOXA3 was detected in the precipitates of anti-HOXC10 in ESCC cells, which indicated that HOXC10 interacts with FOXA3 in ESCC cells (Fig. 4H). In addition, we assessed the impact of HOXC10 deficiency on FOXA3 levels, and HOXC10 deficiency caused a significant reduction in FOXA3 protein levels (Fig. 4I). In summary, we discovered that HOXC10 binds to FOXA3 in ESCC cells and increases the expression of FOXA3.

### 3.5 HOXC10 enhances ESCC cell malignancy by regulating FOXA3

**Fig. 5:**
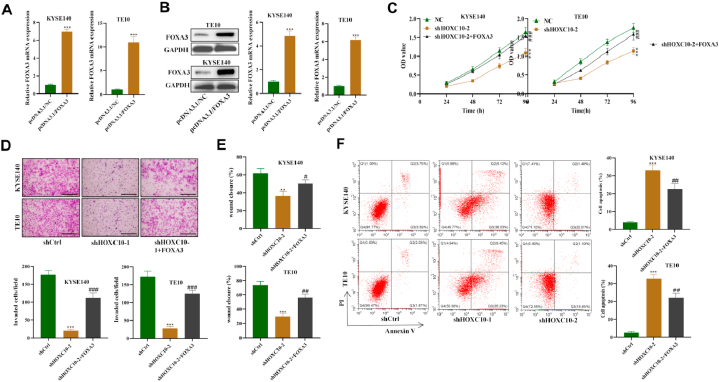
HOXC10 enhances ESCC cell malignancy by upregulating FOXA3. (A) qRT-PCR and (B) Western blot measured FOXA3 overexpression efficacy in ESCC cells. (C) ESCC cell after silencing HOXC10 and/or overexpressing FOXA3 was evaluated using CCK-8 assays. (D) Transwell and (E) wound scratch assays were used to assess invasiveness and migrating capacity of ESCC cells. (F) Flow cytometry was used toexamine apoptosis in ESCC cells after the indicated transfections. All experiments were triplicated. Scale bar = 50 μm **P < 0.01, ***P < 0.001; *###P < 0.001*.

Then we further investigated whether FOXA3 was implicated in the HOXC10-mediated regulation of ESCC cell malignancy. The overexpression efficacy of FOXA3 was confirmed in ESCC cells (Fig. 5A and B). The viability of ESCC cells reduced by silencing HOXC10 was reversed after overexpressing FOXA3 (Fig. 5C). FOXA3 overexpression was also demonstrated to counteract the HOXC10 silencing-induced suppression on the migration ability and invasiveness of ESCC cells (Fig. 5D and E). Moreover, we found that the elevated apoptotic rate of ESCC cells was significantly reduced by the transfection of FOXA3 overexpression plasmids (Fig. 5F). Overall, HOXC10 facilitates ESCC cell growth, migration, and invasion abilities while suppressing apoptosis by upregulating FOXA3.

### 3.6 HOXC10 suppresses ESCC tumorigenesis *in vivo*

**Fig. 6:**
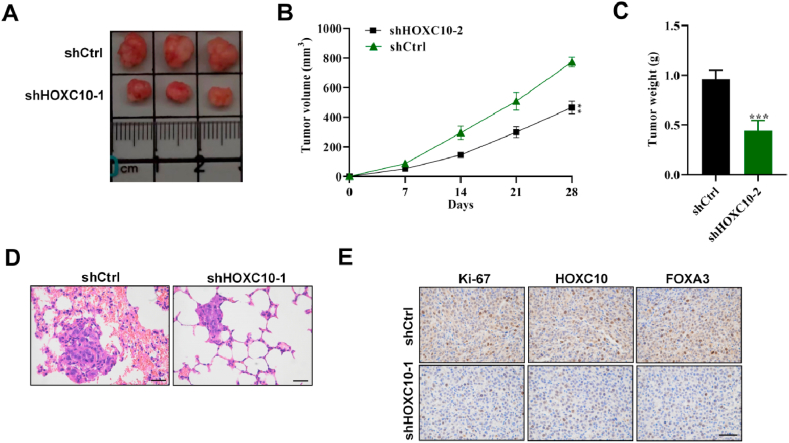
Knockdown of HOXC10 suppresses ESCC tumorigenesis *in vivo*. (A) The murine tumor images in the shCtrl and shHOXC10-1 groups (n = 5). (B) Mouse tumor size curves and (C) weight in the two groups (n = 5). (D) HE staining was conducted to evaluate the morphological changes in mouse tumor tissues. (E) IHC assays were used to measure the protein expression of Ki-67, HOXC10, and FOXA3 in mouse tumors. All experiments were triplicated. Scale bar = 50 μm **P < 0.01, ***P < 0.001.

The impact of HOXC10 on ESCC tumor growth *in vivo* was examined in tumor-bearing mouse models. As exhibited in Fig. 6A and B, the tumor size was smaller in the shHOXC10-1 group and the growth rate was reduced by HOXC10 deficiency. Similarly, the tumor weight showed a significant reduction in the shHOXC10-1 group relative to the shCtrl group (Fig. 6C). According to the results of H&E staining, the morphological changes of lung metastases were alleviated after silencing HOXC10 relative to the shCtrl group (Fig. 6D). IHC assays showed that the protein levels of Ki67, HOXC10, and FOXA3 were significantly reduced in the sh-HOXC10 group. This indicates that HOXC10 regulates ESCC tumor cell proliferation *in vivo* (Fig. 6E). The aforementioned findings demonstrated in vitro mouse models that Hoxc10 can hasten the development of esophageal squamous cell carcinoma.

### 3.7 HOXC10 upregulates FOXA3 to activate the MAPK signaling in ESCC cells

**Fig. 7:**
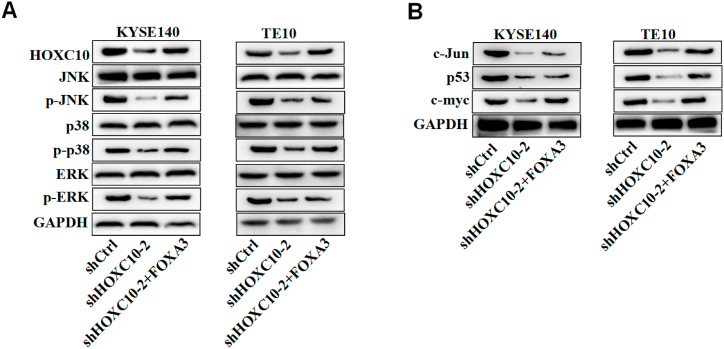
HOXC10 upregulates FOXA3 to activate the MAPK signaling in ESCC cells. (A) Western blot was used to measure the protein levels of HOXC10, FOXA3, and key proteins on the MAPK pathway in transfected ESCC cells. (B) The protein expression of MAPK downstream targets in transfected ESCC cells. All experiments were triplicated.

The MAPK pathway has been revealed to be implicated in the regulation of cell events in the development of ESCC [28]. HOXC10 has been reported to activate the MAPK pathway to regulate cancer proliferation and metastasis [29]. Then we further analyzed whether HOXC10 regulates the MAPK pathway in ESCC. The protein levels of HOXC10, FOXA3, p-JNK, p-p38, and pERK showed a significant reduction in the sh-HOXC10 groups (Fig. 7A). In addition, we also examined the expression of downstream targets of the MAPK pathway. Consistently, silencing HOXC10 caused a dramatic decline in the expression of c-myc, P53, and c-Jun proteins (Fig. 7B). Moreover, we found that the overexpression of FOXA3 in ESCC cells had a counteractive effect against the HOXC10-induced inactivation of the MAPK pathway, which suggested that HOXC10 promotes the activation of MAPK signaling in ESCC cells by regulating FOXA3.

## 4 Discussion

The overall prognosis in ESCC patients remains disappointing partly due to late diagnosis and limited targeted therapy [[30], [31], [32]]. In this study, we utilized an Agilent mRNA single-channel gene expression chip to explore the gene expression profile in five paraffin-embedded ESCC surgical samples and adjacent NTs, and identified differentially expressed genes. HOXC10 mRNA levels in the ESCC samples were approximately 12-fold higher than in the adjacent NTs. Further analysis revealed that the high HOXC10 levels were related to unfavorable clinical outcomes in ESCC patients and underlied its potential for ESCC prognosis.

HOXC10, a highly conserved transcription factor, is expressed at low levels to maintain normal physiological function in most adult cells. However, up-regulation of HOXC10 has been found in many types of human malignant solid tumors that occur in the head and neck, lung, breast, stomach, liver, and ovary. It has been reported to promote tumorigenesis through oncogenic pathways, angiogenesis, release of inflammatory cytokines, immunosuppressive effects, and the activation of epithelial mesenchymal transition (EMT)-related genes [14,16,19,[33], [34], [35]]. The results of these studies indicate that HOXC10 may play an oncogenic role in driving tumorigenesis. Furthermore, a previous study has shown that elevated expression of HOXC10 facilitates cell proliferation and enhances chemoradiotherapy resistance in ESCC [20]. Consistently, in our study, we also found that HOXC10 was upregulated in ESCC and correlated with poor OS as well as DFS in ESCC patients. The deficiency of HOXC10 suppressed the growth, migration capacity, and invasiveness of ESCC cells. It also induced an elevation in ESCC cell apoptosis and inhibited tumorigenesis in tumor-bearing mice. These findings suggest that HOXC10 has suggesting the oncogenic effects in ESCC. Furthermore, the high expression of HOXC10 mRNA, along with cell differentiation and advanced clinical stages, was found to be an independent prognostic factor for ESCC. The data further indicated that HOXC10 could be a promising diagnostic and prognostic marker for ESCC.

Previous literature suggested that HOXC10 acts mainly through upregulating ERBB3 and activating the PI3K/AKT pathway to promote ESCC cell growth [20]. This present study identifies another molecular pathway in the regulation of ESCC cell growth by HOXC10. Our research focuses on FOXA3, the members of forkhead box (Fox) proteins. The members of this family are transcription factors (TFs) sharing homology in the forkhead DNA binding domain and indicated to be crucially involved in cell growth, apoptosis, as well as cell cycle process [36]. FOXA3 has been reported to act as a cancer promoter or anti-cancer gene in various malignancies, including hepatocellular carcinoma, cholangiocarcinoma, and esophageal cancer [[23], [24], [25]]. A study also indicates that FOXA3 is deregulated in lung cancer and that high FOXA3 expression is linked to adverse overall survival, suggesting FOXA3 can serve as a prognostic biomarker for lung cancer patients [37]. In our work, we identified the increase in FOXA3 levels in ESCC tumor samples and cells. The GEPIA and STRING databases indicated a positive correlation between FOXA3 and HOXC10 expression, which was further confirmed by the PCR analysis in our study. Moreover, we found that HOXC10 interacted with FOXA3 to upregulate FOXA3 expression in ESCC cells. Rescue assays further demonstrated that FOXA3 overexpression reversed the HOXC10-silencing induced suppression on the proliferative capacity, migration, invasiveness and apoptosis of ESCC cells, which indicated that HOXC10 regulated ESCC cell malignancy at least partially through regulating FOXA3 expression. Therefore, our study further confirms that HOXC10 plays a crucial role in tumor progression in ESCC and provides a new avenue to understand tumor malignancy in esophageal squamous cell carcinoma.

MAPK pathways regulate all aspects of life and are frequently altered in cancer progression [38,39]. Increasing evidence has revealed that this signaling regulates cellular processes such as cell proliferation, differentiation, mitosis, cell survival, apoptosis as well as metastasis [[40], [41], [42]]. HOXC10 is reported to accelerate the proliferative capacity and metastasis in gastric cancer by activating the MAPK signaling pathway [29]. The three MAPK pathways that have received the most research attention are p38 kinase, ERK, and stress-activated protein kinase (SAPK)/JNK [43]. In this study, it was observed that the inhibition of HOXC10 resulted in a decrease in JNK, p38, and ERK in ESCC cells. This effect was found to be reversed by overexpressing FOXA3, suggesting that HOXC10 plays a role in activating the MAPK pathway by regulating FOXA3 in ESCC cells. The obtained results exhibited conformity with the prior research outcomes. This means that HOXC10's regulation over the MAPK signaling pathway was also verified in ESCCs. Western blotting was used to evaluate the MAPK signaling pathway; nevertheless, there is currently no information available regarding the kinases that are associated with HOXC10. We exclusively concentrated on investigating the classical MAPK pathway and did not validate the non-classical MAPK pathway or its distinct downstream targets. Further investigation is still necessary for all of the aforementioned factors. In conclusion, the upregulation of FOXA3 through the MAPK signaling pathway by HOXC10 has been found promote cell proliferation, migration, invasiveness, and inhibit cell apoptosis in ESCC. A significant correlation has been observed between elevated levels of HOXC10 and unfavorable prognosis in patients with ESCC. Consequently, HOXC10 has emerged as a potential diagnostic and prognostic marker with promising implications in the field of ESCC. However, it is imperative to conduct studies with larger sample sizes in order to establish a conclusive and definitive conclusion. The results of our study have the potential to offer novel perspectives on the application of targeted therapy for ESCC.

## Ethics approval

The study was approved by the Ethics Committee of the Affiliated Wuxi People's Hospital of 10.13039/501100007289Nanjing Medical University, Wuxi People's Hospital, Wuxi Medical Center, 10.13039/501100007289Nanjing Medical University (Approval No: KY23053).

## Availability of data and materials

The data that support the findings of this study are available from the corresponding author upon reasonable request.

## Funding

Not applicable.

## CRediT authorship contribution statement

**Xiaoting He:** Writing – review & editing, Writing – original draft. **Huiyu Wang:** Methodology, Investigation. **Runjie Wang:** Methodology, Investigation. **Yuting Li:** Validation, Formal analysis. **Suqing Li:** Visualization, Validation. **Xiufeng Cao:** Project administration, Conceptualization. **Junying Xu:** Project administration, Conceptualization.

## Declaration of competing interest

The authors declare that they have no known competing financial interests or personal relationships that could have appeared to influence the work reported in this paper.
